# "The Hospital" Nursing Mirror

**Published:** 1897-10-23

**Authors:** 


### "The Hospital" Nursing Mirror.

Being the Nursing Section of "The Hospital."

[Contributions for this Section of "The Hospital" should be addressed to the Editor, The Hospital, 28 & 29, Southampton Street, Strand, London, W.C., and should have the word "Nursing" plainly written in left-hand top corner of the envelope.]

### News from the Nursing World.

### THE QUEEN'S GIFTS AT THE IMPERIAL INSTITUTE.

An afternoon is well spent in visiting the unique exhibition now on view at the Imperial Institute. There the gifts from the kings of the earth are to be seen beside the simple handiwork of Her Majesty's humbler subjects, and one and all tell the same tale of loyal devotion to one who has been a noble and beloved monarch for sixty long years. The personal gifts are contained in one small case, but that case is ablaze with jewels. The Shah's portrait, too, is in a gold frame heavy with gems. The Emperors of Japan and China have sent gifts worthy of the occasion; the needlework in the screens is simply marvellous, and the carving of the wood beautiful. The number of addresses, though gratifying to the nation's pride, are too numerous to enable the spectator to appreciate the wealth of material and workmanship lavished upon them. The exhibition is most popular, and will doubtless bring a substantial addition to the Prince of Wales's Fund.

### TYPHOID AT UNIVERSITY COLLEGE HOSPITAL.

The attack which so lamentably occurred amongst the nurses and maids at University College Hospital has been traced to a temporary water supply, necessitated by the encroachment of the buildings of the new hospital, given by Sir Blundell Maple, upon those now occupied. The water has been disconnected, the patients carefully nursed, and the plague stayed. The type of disease is also very mild, and when we visited the hospital no complications had set in, and there was every hope that there would be no fatal cases. The stricken nurses have been accommodated in three cheery wards, made bright with flowers sent by sympathisers and wellwishers. Typhoid, although a dangerous complaint, is one that can be stamped out as soon as the necessary precautions are taken, and it is entirely the fault of the nurse if the disease is spread from patient to patient.

### MEMBERS OF PARLIAMENT AT PRINCE ALFRED HOSPITAL, NEW SOUTH WALES.

This hospital takes first rank amongst colonial hospitals, and from the description given of it, on the occasion of the visit of members of the Legislative Assembly, it is second to few in the perfection of its equipment. The nurses' home is most comfortable; it is tastefully furnished and adorned, the ladies of Sydney having painted pictures for the drawing-room walls. The dining-room is arranged with a number of dainty, small tables. Dr. Graham, in a speech after tea, explained the use and work of hospitals, and commented on the growth of medical science and the discoveries of Lord Lister. He dwelt upon the value of the trained nurse, and pointed out how much care is now exercised to eliminate unnecessary drudgery from her work; comparing the lot of a hospital nurse favourably with that of her private sister. He also impressed on those present the importance of making some provision for age, and urged the promotion of a movement similar to the Royal National Pension Fund in England. Dr. Graham is apparently unaware that the National Pension Fund is imperial in its scope, and that the nurses of Australia are eligible as members.

### THE HOUSEMAID NURSE.

It is always interesting to find our comments quoted, but slightly distressing when our sayings are perverted and things are attributed to us which we have not said. The *Charlotte Medical Journal* has the following: "There is a growing tendency in England to discard trained nurses, especially in private practice. The *Practitioner* has said many things about them that are far from complimentary. In the July number it quotes from The Hospital as follows: 'Many medical men, and those of the widest experience and highest authority, will confess that for private nursing they prefer the much abused "housemaid" nurse, and that is a view which will be cordially echoed by those of the public who have suffered at the hand of her social superior, the "lady nurse." It is a self-evident fact that ladies do not make the best private nurses, and one which few who have had experience in the management of a private nursing institution will deny.' The *Practitioner*, on its own account, says: 'Many people will continue to prefer the ministrations of an intelligent woman who does not disdain to put her hand to anything that will make them comfortable, and who, if she has not the accomplishments, is also free from the affectations of a lady nurse.'" But we never said anything to suggest that there was "a growing tendency in England to discard trained nurses." The "housemaid" nurse and the "lady" nurse are equally trained and equally capable. The difference is merely one of social habits and associations, which cause the first to be more fitted for one post and the second for another.

### A HARVEST FESTIVAL.

The harvest festival service was held on Wednesday evening last in the chapel of the National Hospital for the Paralysed and Epileptic (Albany Memorial), Queen Square, when an address was delivered by the Ven. the Archdeacon of Middlesex. The exceedingly tasteful decorations had been carried out by the Lady Superintendent (Miss Rachel F. Tweed), the Assistant Matron (Miss Daunt), and helpers, it being an interesting fact that nearly the whole of the flowers and foliage employed had been supplied from the gardens of the New Convalescent Home at Finchley, including a large consignment of everlasting flowers (helichrysum), which will remain available for the wards during the winter.

### THE GERMAN RED CROSS SOCIETY.

At the third annual meeting of the German Red Cross Society, which took place in Berlin on October 1st, many influential members were present at both the morning and afternoon sittings, amongst others: H.R.H. Princess Ludwig of Battenberg, Princess

Victoria of Hesse, the president of the Alice Confederation of Women, Dr. Niedhardt, Herr Chuchal, president of the Central Committee of the Red Cross, and Prince Nikolas of Nassau, president of the Wiesbaden Union of the Red Cross. An interesting paper on the "Protection of Nurses from Infection" was read by Dr. Theodor Rumpel, senior physician of the New General Hospital in Hamburg. In view of the great importance of this subject, one of the three members of the existing Commission of Experts was authorised to lay before the next annual meeting a short paper on precautions against infection, for the use of the sisters. Dr. Rumpel was chosen president of the commission.

### THE HUMOURS OF BUMBLEDOM.

The Lambeth Board of Guardians, having found the present condition of the nursing in the infirmary harmonious, have resolved to change all that. Instead, therefore, of appointing an assistant matron in place of Miss Tomkins, they, by eleven votes to six, have vetoed the proposal, and have decided to appoint a storekeeper and cutter-out. This new arrangement leaves the probationers without supervision at night; and as there have been occasions on which the young women have sat at their windows and waved hands to the passers-by, we cannot but think that more and not less supervision is necessary, unless the guardians wish to have their proceedings enlivened by many a budding scandal.

### GAGGED OR KIDNAPPED.

The grandiloquent mandate which claims to come from the Incorporated Medical Practitioners' Association, calling for a public inquiry into the affairs of the Royal British Nursing Association, is honoured, in an evening paper, with the sensational heading, "'Gagging' the Nurses." This, of course, is intended as descriptive of the condition of the unfortunate nurse members of the Association at the present time. But we fear matters are worse than this. A meeting, reported to be held on their behalf by what is called the Members' Rights Defence Association, took place in the St. Martin's Town Hall. But, in spite of the existence of upwards of 3,000 nurse members, who had the opportunity of coming forward and asserting their rights and airing their wrongs, none but Nurse Beatty actively supported the promoters. It would be reasonable, therefore, to conclude that the whole Association has not only been gagged, but kidnapped. For if nurse members of the Royal British Nurses' Association were in sympathy with Mrs. Bedford Fenwick's movement on their behalf, they would have attended the meeting to support their champions. As a matter of fact they were conspicuous by their absence.

### A DISMAL OUTLOOK.

The officers of the Chelsea Hospital for Women have a serious bone to pick with the Jewish authorities, who are responsible for the condition of the disused burial-ground lying beside it. Owing to the situation of the hospital in the midst of busy thoroughfares, this open space, if decently kept, would be a great boon to the patients and nursing staff, especially as roomy balconies are built at this end of the long central corridors of the different storeys. The green headstones shouldering each other in melancholy and disorderly rows, the rank grass and weeds ornamented with scraps of waste paper suggest uncompromising misery, and this to women in a hyper-sensitive, nervous state is very depressing. The case has been represented at Jewish headquarters, the Chief Rabbi has been spoken to, and invited to view the desert, but all has been without profit. It is indeed difficult to imagine a reason why this ground is not converted into a "pleasant place," as refreshing to the enforced spectators as it is now the reverse. Reverence for the dead it cannot be, unless dilapidated gravestones and rank undergrowth denote reverence. It can hardly be indifference to the sufferings of the sick, for our Jewish fellow-citizens have shown themselves liberal and sympathetic aiders and abettors in all charitable undertakings. It must, therefore, be a careless oversight on the part of busy men. If such be the case, we urge that the state of this cemetery is not a little thing. There is an average of 15 operations per week at the hospital, and the nurses are 16 in number. Both patients and nurses can be refreshed and strengthened by a trifling expenditure, which would be as much for the honour of the dead as for the welfare of the living.

### NURSES AT MAIDSTONE.

Nursing uniforms of every description are seen in Maidstone just now, and they provoke neither comment nor criticism, for they are worn by earnest, purposeful women. The colour of a cloak or the length of a veil seems of little importance in the face of a typhoid epidemic. There are other workers in the town who, as watchers and helpers, are valuable, nor trespass on the trained nurses' special, responsible duties. Much kind aid is given by ladies resident in the town and neighbourhood, and there is plenty of work for all. In another column our "Special Correspondent" points out that all *nurses* (whether belonging to the Nurses' Co-operation, or any other accredited society) are able to get such necessaries as their work requires from "The Nurses' Stores." The large schoolroom in King Street has been kindly placed at Miss Gethen's service, and there Miss Maule gives out what is required from ten to five o'clock daily.

### SHORT ITEMS.

Several ladies connected with Shropshire are organising a Benefit Nursing Association, in order to supply that county with cottage nurses. The Duchess of Sutherland, Lady Meyrick, Lady Ashburnham, and Lady Priestley are amongst the number.—The Mile End Guardians on the School Committee, after making a searching inquiry into the charges of cruelty against a nurse in their employ, were equally divided on one point only, viz., should the nurse be dismissed or asked to resign. The chairman's vote saved the nurse. It seems a pity that when a nurse has been found guilty of cruelty to children she could not be kicked out of the profession. Dismissing her from one post but leaves her at liberty to exercise her cruelty elsewhere, whilst asking for her resignation is much too mild a penalty for her offence.—Nurse Kane, of the Stockport Workhouse, was presented with a handsome prayer-book with this inscription: "Presented to Susannah Kane by the patients of the convalescent ward of the Stockport Workhouse as a small token of their esteem on her retiring into private life."—The Countess of Warwick presided over the second annual meeting of the Dunmow and District Nursing Association. It was arranged that a nurse should be lent to neighbouring parishes in cases of emergency on their paying a yearly subscription of not less than £1 1s.—The India Office have applied to the R.B.N.A. to supply the Government with fully-trained nurses willing to volunteer for the plague duty in Bombay.

### Practical Aspects of a Nurse's Life.

By Sister Grace.

### VIII.—PROBATIONERS IN SURGICAL WARDS.

### Obstetric Work.

The subject of obstetric work is so vast, and to a great degree stands so much apart from other nursing, that it would occupy too much space to enter upon here. I can, therefore, only glance at it casually before passing on to other subjects. Let me draw your attention to the necessity of the most conscientious regard to all the details, especially surgical cleanliness. This is essential in all surgical work, but if possible it is more imperative in the obstetric wards.

Before entering on your day's work, be careful that all your clothes, upper and under, are spotless and frequently changed, and that your hands and nails are immaculate. Another thing I would impress upon young nurses when they first begin work of this kind is to beware of any tendency to disregard the dictates of womanly delicacy in what must always be a trying part of a nurse's work; in some natures familiarity breeds contempt, and what at first brings with it feelings of discomfort soon ceases to be anything but routine; endeavour always to remember that the patients have feelings to be considered; try to place yourself in their painful position, and you will see how greatly they are helped and comforted by having a nurse of delicacy and refinement. And if you should meet with patients who are unfortunately careless of personal propriety and delicacy, it must be your aim, not to rebuke and lecture them, but by your own modest and careful deportment to show them the beauty of true womanliness. There is much within a nurse's power in this way in all departments of her work. You will be thrown much with students and doctors generally; be careful that they do not entertain a lower opinion of your sex through your example. A nurse's influence is like a stone thrown into a pond, where the widening circles it causes are at last lost to view. It is a serious and solemn thought; do not let it pass from your mind.

### Abdominal Sections.

All these cases, and others which, without being (strictly speaking) abdominal sections, may be reckoned under the same head as requiring much the same treatment, are infinite in their variety, since abdominal surgery has attained such popularity, and are all serious. Different arrangements prevail in hospitals as to the nursing; some keep them in the general wards, and others consider it necessary to isolate them from all contaminating influences. Abdominal sections are usually more serious with men than with women, because they are, generally speaking, undertaken for more serious conditions, such as intestinal obstructions and growths caused by malignant disease, &c., whereas many cases in the women's wards undertaken for the removal of tumours (not necessarily malignant) are frequently hopeful, and often entirely successful. It is not my province to explain to you the nature of operations, but simply to show the best way to nurse the case before and after.

See that the patient is scrupulously clean by the administration of a carbolic bath, or if too ill to endure that, wash him thoroughly in bed. See that the bowels have acted freely, and that an enema has been given; it is imperative that a thorough clearance should be made before operation, because it is undesirable that there should be any action of the bowels for several days. It will not, probably, be your duty to see to so important a matter as the arrangement of the patient's food, but I may tell you that it is inadvisable to give solid food for some hours before the administration of chloroform; vomiting may occur, and if the patient throws up solid food in his unconscious condition, it is likely to lodge in the throat, and he will be choked. At the same time, I think it is a great error to allow patients to undergo an operation when they have been a long time without nourishment, and in my experience it conduces to sickness. I find it a good plan (when no orders are given) to administer about 8 ozs. of strong beef tea three hours before the patient goes to the theatre. The preparation of the bed for all cases of abdominal surgery, and for operations to the lower part of the body, is important, as well as careful attention to macintoshes and draw-sheets. The bed-clothes must be so arranged as to open across the middle, for the convenience of dressings, &c. . . . This avoids the danger of cold, and prevents the patient having a painful feeling of unnecessary exposure. The thighs must be supported on a pillow, to remove all strain on the abdominal muscles, and as he must lie some time on his back, it is well to have the shirts entirely open to the neck behind, to avoid creases and facilitate changing.

In the case of women, the catheter must be passed, to avoid the slightest movement, and do not forget to arrange the hair in two plaits, so that there is no lump at the back of the head. Treatment differs much as to the administration of nourishment; some doctors will not permit anything to be given by the mouth for 24 hours, others allow a little liquid to be thus taken within a short time if there is no sickness. I incline to this treatment, followed judiciously. You will understand that in all abdominal surgery vomiting is very serious; it may induce hæmorrhage, or split stitches from the wound, or complicate matters in many ways.

Ice in small quantities will sometimes conduce to arresting sickness, but it has a tendency to increase the thirst already experienced, and if the quantity is great, colicky pains will be induced. The closest watchfulness is necessary in these cases. If the patient should during sleep turn on his side the result may be fatal; hours for administering nutritive enemata must be closely adhered to, and no single care relaxed as convalescence commences. Be very careful about cleanliness; the position of the patient makes attention where it is most necessary easy and simple; everything can be done without disturbance or discomfort. Keep the patient lightly but warmly covered, and the room at an even temperature. If any sudden pallor or greyness appear give notice of it at once; it is often a sign of internal hæmorrhage, a danger always to be feared if there is much vomiting.

### A Contrast.

The manner in which some nursing associations exploit the earnings of the private nurses for the benefit of the district nurse was illustrated in the case of the nursing association of Croydon. A happy contrast is afforded by the custom obtaining at the All Saints Home for Trained Nurses, in Fitzroy Street. This association aims at nursing all classes, the poorer as well as the richer, and whilst the latter are charged full fees, the former are nursed at fees regulated by the amount of their income. By the rules of the Home, a percentage of 2s. 6d. in every pound is paid the nurse on duty, and as the full fee is £2 2s. a week, the nurse receives 5s. a week in addition to her fixed salary. It is satisfactory to learn that each nurse has her full percentage, without regard to the fee paid to the home. This is only strict justice; but justice is not always strictly dealt out to the nurse, so that when an instance occurs it is very pleasant to be able to record it.

### Post-Graduate Clinics for Nurses.

By a Trained Nurse.

XXXIV.—THE DIGESTION OF SOLIDS AND FLUIDS.

It would be well to draw attention to the variations one meets with in conditions of nausea and vomiting, as it is most important for the nurse to distinguish between the one kind and the other; for there is the nausea and vomiting of repletion, or that arising from disordered digestion, or from the fact that some article of diet has "disagreed."

On the other hand, in nervous diseases, and in many wasting illnesses, the nausea of exhaustion may sometimes be mistaken for the nausea of a disordered stomach. An exhausted patient whose nourishment is not administered sufficiently often, frequently exhibits all the distaste and apparent loathing of the food brought to him as is exhibited by persons suffering from biliousness and true sickness; and it is sometimes extremely difficult for him to appreciate the fact that this loathing of the smell and sight of the food presented to him may be in itself a sign that his exhausted condition calls for nourishment. The general indications of the patient should lead the observant nurse to distinguish the difference between the two conditions. And the main index to this state of things rests in the fact that the nausea of exhaustion is relieved by food, the nausea of repletion will be increased after nourishment.

Many patients complain each morning of feeling so "deathly sick"; they are convinced that they will be unable to take any breakfast. Now this morning sickness usually arises from exhaustion. And sometimes the condition is aggravated by the fact that nurses do not always appreciate the grave importance of early morning feeding. It is so common to hear nurses in private practice priding themselves on "only giving their patients the very lightest food during the night." It would be difficult to make a graver mistake. For, as a matter of fact, it is more important that the most nourishing food taken during the twenty-four hours should be given in the night and early morning. The vitality is then lowered, all the energies are at their least ebb, and more fuel is needed to keep the sparks of life together. For the same reason that more fire is necessary at night, and that the most elementary nurses are taught to "draw an extra blanket over the patient" at about two a.m., so more food is necessary. It is not altogether a question of the colder atmosphere calling for more fire and an additional blanket. It is largely a question of depressed vitality. Special stress, therefore, should be laid on night and early morning feeding, and—other conditions being favourable—warm or hot food is preferable as affording more stimulation and energy, and for the reason that hot drinks promote sleep and digestion, while cold foods are apt to keep patients wakeful.

Where nausea is present more or less constantly as a result of an exhausted condition on the part of the patient, it often proves most valuable in practice, if the patient be "on stimulants," to give a small quantity of alcohol about ten minutes before a meal, or any food calling for digestive effort, is given. It is surprising how well an ounce of wine or two drachms of whisky in a small quantity of cold Apollinaris or Seltzer before a meal acts on the digestive power. The effect, of course, is to increase the vascularity of the stomach, and to cause an added flow of gastric juice. A small glass of champagne—with or without an aerated water—given in the same way acts as an effective check on the nausea of exhaustion, and enables the patient to get up "quite an appetite" for more satisfactory nutriment. If the patient be not allowed alcohol, then a substitute must be found to act as a pick-me-up to the stomach in preparation for more substantial food. Two teaspoons of Valentine's meat juice in half a wineglass of cold aerated water will prove most useful; or a dessert spoonful of frozen beef essence may be given. As a slight digression, I would beg nurses to remember never to give meat jellies or essences without previously putting them on ice. I retain after many years the most gruesome recollections during a serious attack of diphtheria, of the sickly half-warm and altogether nauseating hospital beef-essence, whose administration was an "every two-hours'" penance. It would have been easy since ice was so plentiful to have given the patients their meat-essence in a cold, refreshing, and appetising form. The real secret why nearly all patients dislike meat-essence so much and why they leave a large proportion of their prescribed "dose" on their plates is because it is generally given lukewarm. I have never known a patient to reject frozen meat-essence. It is most important, even in the nausea of exhaustion, that small quantities of food should be given at a time, and very frequently. Even though the nausea arise from exhaustion and the need of food, the stomach under these conditions needs gentle treatment, and to overload it, with the idea that because the need of food is great the power of assimilating it is also great, will result in seriously hampering the digestive capacity.

A generality found often in nursing and food books is the statement that fluid foods are more easily digested and assimilated than are solids. Now this rule has many and wide exceptions. In some forms of gastric disturbance, as for instance in atonic or flatulent dyspepsia, it is frequently found that small quantities of light solid food—this sounds a little like an Irish bull, but it is eminently practical—is much more easily tolerated than are liquids.

So that the nurse must exercise her tact and employ a careful watchfulness as to whether a rather dry diet suits her patient better than a liquid one. Of course this advice is meant for those cases where the diet is a matter of option. In many cases, of course, a fluid diet must be adopted for other reasons, although from a purely digestive point of view solids might better suit the stomach.

Every nurse must, if her experience be at all large, have come across many cases in which a few spoonsful of delicately minced chop have been retained, where every fluid form of food has been rejected by an irritable stomach. It seems almost superfluous—but in face of the dietetic ignorance which our system of hospital training renders possible among very highly educated nurses—to remind all who have the responsibility of dieting the sick, that twice-cooked meat must never be set before a patient. To do this might be described as the worst sacrilege to which the sick-room could be subject. Therefore, if a little fresh solid meat be tried in cases of the bad digestion of fluids, it must be just freshly cooked, and not that most hopeless of all compounds, from the dietetic point of view, "re-warmed" mutton or chicken.

It is a curious fact that an irritable digestion which may prefer a small amount of solid to fluid foods will in nine cases out of ten deal better with red meat than with white meats. This may be against preconceived theories. But "facts are stubborn things," and I would at all times maintain a faith in the juicy tender part of a chop for an uncertain stomach, rather than in the tenderest piece of chicken. And nurses who find in particular cases that fluid foods do not "sit comfortably on the stomach" should never lose sight of the incontestable fact that fluid foods "thickened" are often much more easily taken. I do not mean "thickened" with corn flour and arrowroot, for these generally prove too heavy, but thickened with rusks, "bread jelly," gelatine, isinglass, powdered biscuits, either plain or malted.

### The Royal British Nurses' Association.

THE quarterly meeting of the General Council of the Royal British Nurses' Association, held at the offices on Friday, the 15th inst.—Mr. T. PICKERING PICK in the chair—was very largely attended.

After Dr. BEDFORD FENWICK had raised the point whether the Council had been properly elected at the last annual meeting,

The HON. TREASURER (Mr. John Langton), in presenting his quarterly report, announced that the action for libel brought by Dr. Bedford Fenwick against Miss De Pledge had been abandoned. Although they would all be glad, on the score of expense, that the action had been withdrawn, yet he was sorry, for if it had been carried to an end people would have been able to see how far the allegations now being freely made against the officials were correct. (Hear, hear.) He formally proposed the adoption of the report.

Mr. F. J. GANT seconded, and the resolution was carried *nem. con.*

The HON. MEDICAL SECRETARY (Mr. E. A. Fardon), submitted the report of the Executive Committee. In doing so he mentioned that the hon. treasurer had again advanced the Association a further loan of £100 to enable it to carry on its work. (Applause.) After referring to the arrangements which were being made to issue the journal of the Association monthly instead of quarterly, the speaker congratulated the secretary, Mrs. Ranken, who was leaving owing to her marriage, on the way in which she had discharged the duties of her office during the time she had been connected with the Association. (Applause.) As to the withdrawal by Dr. Bedford Fenwick from the action which he had brought against Miss De Pledge, the speaker read a letter, dated October 6th, 1897, received from the plaintiff, in which he said he had withdrawn from that action owing to the fact that the recent decision of the Court of Appeal in the case brought by Miss Breay against the Association had shown him that the Association was able to use its funds, as it proposed to do, to defend Miss De Pledge. As the Association had not aggrieved him, and as he felt that if he continued his action it would, under the circumstances mentioned, involve it in heavy expenditure, he felt that he had no alternative but to relinquish the action. Mr. Fardon also referred to the meeting held at St. Martin's Town Hall on the 13th inst., which was called, ostensibly, by a body termed the Members' Rights Defence Committee. He had no knowledge who the persons who constituted that committee were. He had never seen any list of their names, and he therefore could not say whether it was confined to the members of the Association or whether it included others. From the names of some of the speakers at that meeting he would imagine the latter to be the case. He believed Miss Breay was the honorary secretary of that committee, and he supposed it was to that committee that their Association owed the actions against Sir James Crichton Browne and against the Association. Who constituted that committee he did not know, but there was a body of a more responsible character than that committee—the Incorporated Medical Practitioners' Association—which had taken upon itself to prepare and publish a document containing various charges against the officials of the Association. That document was issued in the name of the Central Council of that association, another body whose members' names he could not obtain. It was signed, he knew, by Dr. Hugh Woods, the president of that association, but Dr. Hugh Woods had told him himself that of many of the statements he had no personal knowledge, and that he had only obtained them on hearsay. That being so, he (the speaker) would like to know which of those statements Dr. Woods knew only from hearsay, and whether the other members of the Central Council had also made themselves responsible for statements of which they had no personal knowledge. He thought the Association had a right to know who constituted both the bodies, and also from whom the Central Council had obtained the hearsay particulars on which they had based their accusations against the Association. From one of the reports of that meeting he saw that it had been stated that discussion of the new bye-laws had not been allowed by the officials. He was astonished that Dr. Woods, who was in the chair, should have allowed such a statement to go uncontradicted, as it was within his (Dr. Woods') knowledge that so far from discussion by the committee having been prevented, the bye-laws had been fully discussed at the special meetings held for that purpose, and no member had taken more part in those discussions than Dr. Hugh Woods himself. Another very grave matter was this. In that same document it was stated that the officials (who, as his hearers knew, were men of position in the medical profession, men who, some of them, had for years held an honourable position in that profession) had taken up the Association in order to further their own private ends. Such a statement should not have been made by medical men about medical men, unless they had been able to prove it up to the hilt. (Applause.) In conclusion, he asked Dr. Bedford Fenwick whether it was true that the meetings of this Central Council were held at his own private house, and that they were held there at a time when he had a personal action pending against the editor of the journal of the Association to recover £1,000 damages for an alleged libellous article? He proposed the adoption of the report.

Miss DE PLEDGE, in seconding, thanked the members of the Association for the very kind way they had supported her, for the sympathy they had shown her, and for the help she had derived from every one of them. (Hear, hear.) She also regretted that Dr. Bedford Fenwick's action against her had been withdrawn, for it might have brought to light many things which had been obscured in the past. (Applause.) There were a good many things behind the scenes which the public had only to know in order to find out that the officials were not so black as they were painted. (Applause.) Now that the action had been withdrawn she would like to say that she had not written the article complained of. (Applause.)

Dr. BEZLY THORNE was extremely glad that the Association had been spared the expense of further frivolous and vexatious litigation, but on the other hand he was not sure that it would not have been to the ultimate advantage of the Association if this action had been carried to an issue, because if it had a good many persons, including Dr. Bedford Fenwick and himself, would have been placed in the witness box, and there were many of them there who would have been very glad to have the affairs of that Association investigated by an impartial judge and before an impartial court. (Applause.) In the letter which Dr. Bedford Fenwick had written assigning his reasons for dropping the action he had alluded to his resigning the treasurership. As that was the fourth time that Dr. Bedford Fenwick had done this at council meetings, and as Dr. Bedford Fenwick had never dared to specify to the Council in detail his reasons for resigning, he (the speaker) begged to give notice that at the next meeting of the Council he would propose the following resolution :—

"That having regard to the fact that Dr. Bedford Fenwick has formally called the attention of the General Council to certain reasons which led him to resign the office of treasurer, and to the fact that Dr. Fenwick has never laid those reasons in detail before the General Council, either at the time of his resignation or subsequently, the General Council request the Executive Committee to institute an inquiry into the circumstances referred to in a communication made to that body on the 2nd of March, 1894, into the conduct of the business of the Association during the year preceding that date, and for as long a period subsequent to that date as the continuity of events may appear to require." (Loud applause.)

Dr. BEDFORD FENWICK said that whatever Dr. B.zly Thorne said now he apparently did not feel able to contradict that document at the time it was sent in. Referring to his letter as to withdrawing from the action against Miss De Pledge, he said that members when they read that letter would see that, consistently with his duty to the Association, he had nothing to do but to sacrifice his own desires and so prevent the enormous expense that the Association would have incurred had the action been proceeded with.

Sir JAMES CRICHTON BROWNE said that Dr. Bedford Fenwick's letter was dated October 6th, and the decision of the Court of Appeal, which he (Dr. Bedford Fenwick) stated rendered it necessary for him to abandon the action, was given in June or July. If that was his reason for abandoning the action why had not that letter been written three or four months ago, seeing that as long ago as that Dr. Bedford Fenwick knew that the costs would certainly fall upon the Association? (Applause.) The officials of the Association had been accused of shirking, but in his opinion that term would be much more properly applied to a gentleman who, after holding the threat of an action over a lady's head for a year, shirked it at the last moment. (Applause.)

Dr. WETHERED said that one of the statements made by Dr. Bedford Fenwick in his letter was very misleading, and that was to the effect that, having written to the journal in November, 1896, and the letter not having been printed, Dr. Bedford Fenwick had no course open but to bring an action against Miss De Pledge. As a matter of fact, the threat of the action was brought in October, 1896, before the letter was received by the journal.

Dr. BEDFORD FENWICK said that was not true. (Laughter.) If they read the journal they would see that his letter was received and acknowledged by Miss De Pledge on November 7th. His letter did not appear in the November number, which was issued on the 19th, and it was not until Miss De Pledge did not, when asked to do so, insert it in the February, 1897, issue, that a writ was issued.

The HON. MEDICAL SECRETARY said he held in his hand a letter to Miss De Pledge from Messrs. Mear and Fowler on behalf of Dr. Bedford Fenwick, dated October 15th, 1896——

Dr. BEDFORD FENWICK: I can only say that I have never seen that letter. (Laughter.)

The HON. MEDICAL SECRETARY (continuing): In which it was threatened that unless she signed a written retraction of and an apology for certain statements which appeared in the August number of the journal, a writ would forthwith be issued. That letter, as he had said before, was dated October 15th, 1896, and Dr. Bedford Fenwick's subsequent letter was dated November 6th, 1896. (Applause.) After that letter of October 15th there was no other course but to put the matter in the hands of their solicitors, whose advice had been followed ever since.

The report was shortly afterwards put to the meeting and carried unanimously.

After re-electing the retiring vice-presidents,

Dr. BEZLY THORNE proposed the re-election of the honorary treasurer, the hon. medical secretary, and the hon. nurse secretary. He was sure that the vast majority of the Association placed implicit confidence in those officials, and proof of that statement was supplied by the voting at the annual meeting, when although those who cast aspersions on the officials had three months in which to whip up every possible vote only one-eighth of the ballot papers returned were against the officials. (Applause.)

Mr. J. F. GANT, in seconding, said that after all the whole of the difficulties the Association had had to face arose from one party trying to turn out the other party. (Applause.) Their officials were men of irreproachable character and of high professional reputation, and he did not see what earthly good would be accomplished by turning them out and abandoning the Association to what he would call a tyranny. (Applause.)

The resolution was agreed to *nem. con.*

After electing the Executive Committee for the ensuing year, the proceedings terminated with a vote of thanks to the chairman.

### All Saints' Home for Trained Nurses.

THE outbreak of typhoid fever amongst the nurses and maids at University College Hospital has created so much interest with regard to the sufferers that a short account of the home to which they belong cannot be unacceptable.

About twenty years ago 3, Fitzroy Street, was taken and fitted up as a nurses' home by a member of the Anglican Sisterhood attached to All Saints, Margaret Street. Sister Cecilia is the present sister superior. All the nursing at University College Hospital is under the management of the Sister Superior, and the private staff is also under her control. This has been a great advantage during the present crisis, as the vacancies caused by the nurses' illness have been filled from the private staff. Another advantage is that nurses on the two staffs are from time to time exchanged, giving both opportunities for study and rest.

The training of probationers is carried on entirely in the hospital. The fees in a few cases are remitted, but the arrangement is a private one with the Sister Superior, and no difference is made between the paying and non-paying pupils. The hospital staff is accommodated in the hospital, and those on the private staff at 3, Fitzroy Street. A new nurses' home will be built in the course of the next year or two. It is part of the plan of the new hospital given by Sir Blundell Maple. Then every nurse will have a separate bedroom, and the curtained cubicles now existing will be swept away.

The nurses' hours for duty are from 8.30 a.m. to 9 p.m., with two hours recreation in addition to the time allowed for meals. Once a week each nurse is given a free evening from 6 to 10 p.m., and is permitted, on obtaining permission from the Sister Superior, to spend the night with her friends, returning to her work at 10.30 the following morning. A month's holiday is allowed in the year.

The idea of periodical fastings and other mortifications of the flesh is so firmly associated with religious communities in the public mind that it is satisfactory to know that a liberal table is kept at the home, meat being served thrice daily, and that beyond the facts that nurses must be members of the Established Church, and that care is taken to secure to each one an opportunity of attending Divine worship on Sundays, there are no religious restrictions whatever.

The salary of a nurse on the private staff is £25 a year (with an additional 5s. a week when nursing a case) for the first two years after training. It is then raised to £30 per annum, and later to £35. If a nurse becomes incapacitated by age or illness she receives a pension, and is provided for either at the home or elsewhere. Each case receives individual consideration. A cottage at Eastbourne is fitted up for nurses needing rest and change, and they may spend their holiday there for 5s. a week.

The nurses enjoy the use of an excellent library. The interest on a fund, raised in memory of one of the sisters, supplies new books, and the old ones are kept in repair by an annual subscription of 1s. a year.

The Sister Superior undertakes the care of patients belonging to the poorer middle-class whenever she can afford it. Last year she nursed 12 free cases and 45 at reduced fees. In this she reaches a class very difficult to help, and she has reluctantly been obliged to refuse patients because she could not afford to send out more nurses under these circumstances. In this the public can help her, and to such as are willing to do so she will give full particulars.

We are unable to express an opinion on the nursing arrangements in University College Hospital or upon the justice or otherwise of the statements now current as to the defective food supply, as our representative was informed by the hospital authorities that the disorganisation due to the preparations for rebuilding forbade an inspection.

### Typhoid Fever at Maidstone.

### THE NURSES.

NEVER in the history of nursing has such a gathering of nurses taken place as is at present to be seen at Maidstone. Between sixteen and seventeen hundred persons are there lying stricken with typhoid fever, and nurses have been summoned from far and near to attend to them. They are at work in private houses, in emergency hospitals, and amongst the poor in their homes, and they may now be reckoned by hundreds.

The London Hospital has supplied a band of them in their neat "private nurses'" uniform, and the Bond Street Association has sent down some of its staff. Princess Christian's own nurses were mentioned in THE HOSPITAL last week, and besides them there are "registered" and "chartered" nurses from their several institutions. The largest contingent is from the Nurses' Co-operation, 8, New Cavendish Street, which has already been called upon to send nearly a hundred members of its staff. These nurses are representatives of a vast number of training schools, both metropolitan and provincial.

The housing of such an influx of workers has taxed the resources of the town, and various public institutions have been requisitioned for dormitories. Many nurses are being hospitably lodged by residents in the town and its outskirts. These are lucky, for they are made very welcome guests, and enjoy every consideration. The less comfortably housed nurses think lightly of their drawbacks, and there is a great tendency amongst all to make the best of things.

Brought face to face with the awful sorrow and suffering of the town, personal trials fade into insignificance. All the nurses are working heart and soul for the sick, rejoicing in each sign of recovery which manifests itself among their patients, and doing bravely and well. Every nurse who reads THE HOSPITAL must sympathise with the work now going on at Maidstone, but only those who have seen it can picture accurately the terrible condition of things. The doctors are at work early and late, and their rest and meals must be compressed into abnormally short periods, for they are apparently always on duty. The hours of the nurses vary a little, but are necessarily long and busy ones. They get their meals away from their work—in restaurants, at the Howard de Walden Institute, and other public places. The hospitals, which have been furnished and opened in such rapid succession, will be described next week, for they deserve an article to themselves. Miss Plowman, who is supervising the whole of the emergency nursing department, has her headquarters at the Station Hospital, and visits the others frequently.

The Corn Exchange has been converted into a huge depôt for clothes and other things sent as gifts for the poor of Maidstone. Orders for these things are signed by clergy and others. Sad are the messages which accompany some of these orders when the requisition is for "black clothes" that the petitioner may be decently clad for the funeral next day of parent or near relative.

Beef tea, milk, and broth are supplied for the sick, and many other things which the nature of the illness requires are forthcoming.

In addition to the central stores at the Corn Exchange a little emergency store of nursing appliances of every kind has been established, and has already proved of great value to the sick by facilitating the work of the actual nursing. This store is merely supplementary to the others, and is managed exclusively for nurses.

In the first place a room was hired at the Temperance Hotel to receive gifts sent for the use of the Co-operation nurses, and to serve as a centre for their distribution. In the course of a few days the response to an appeal made by the secretary through the Press was so liberal that the first room became quite inadequate for the purpose of storage. A large school-room has now been lent, and there all strictly nursing necessaries are supplied as well as linen, clothes, &c. All nurses working in the town are made welcome here, and their wants receive prompt attention; and if these include articles not in the stores, and which are really required by doctors or patients for the proper nursing of the sick, the nurse is allowed to requisition them, and a telegram or letter is despatched by Miss Maule, who is in charge, assisted by Nurse Emily Elliot. They evolve order out of the heterogeneous mass of parcels, and send away the nurses well-contented with most bulky parcels of useful things for their patients. The general good-fellowship which has been established amongst all the nurses working in the town in this epidemic is fostered by the knowledge that they are one and all made equally welcome, and their wants freely supplied by the Nurses' Store.

All kinds of warm clothes, especially for boys and children, are in great demand, and will be gratefully acknowledged by H. F. Gethen, the Nurses' Stores, Maidstone. Contributions for the purchase of the many expensive accessories which the nursing of typhoid fever demands will also be gladly received at the same address, and by Miss Lamport, 52, St. John's Wood Road, London.

### District and Parish Nurses.

### A LETTER FROM MISS TWINING.

WE reprint a letter to the *Guardian* of October 6th, as it bears upon a very practical point:—

"Having been connected with the District Nursing Association, first begun in London in 1875, during this whole time, I should like to say in reply to your correspondent, that I and many others have long desired that further and more satisfactory efforts should be made towards payments, in some form, from the poor who benefit by the visits of the nurses. From the very first the subject was discussed by the Council in Bloomsbury Square, but it was found impossible to adopt any plan at the beginning of the work, as the system being entirely new it was looked upon with suspicion by the poor, and it required time to accustom them to the idea of ladies visiting them as nurses. But even then, I remember that one of our members, a clergyman near Holborn, objected to the plan as adding another to the too many already existing free medical aids, which he considered pauperising.

"But surely now the time has come when the matter may be reconsidered, and some system decided upon and sanctioned by the central authorities at St. Katherine's, who would naturally be the best qualified to take it up. At present each branch is at liberty to act as seems best to itself, and there is consequently no guide to what is the most desirable plan.

"From the first free-will offerings were made by some grateful patients, but as no demand can be made by the nurses this is a very uncertain system, and some branches announce that the nursing is 'without charge.'

"I believe that a connection with the payments of provident dispensaries has been carried out in London, I am not aware to what extent. Now that over 100 Boards of Guardians subscribe to outside associations for nursing the outdoor poor, the class of the really 'destitute' is provided for, and it therefore seems most desirable that some plan should be devised for payments from the working classes above paupers. When the class of tradespeople has been attended (and the middle-class has been hitherto left without nursing in an even greater degree than the 'poor'), payments according to their means have of course been made.

"Now that the system is spreading everywhere, I earnestly hope that some plan may be devised for preserving the independence of the poor in this matter, whether by 'benefit clubs' or some other way."

### Nurses for the Plague.

During the present year twenty-four nurses have been sent to nurse the plague from the India Office. One party sailed at the end of last week, and another some little time before. In view of the possible necessity to send others, nurses possessing the necessary qualifications may place their names on a register as a volunteer ready to embark from London within a week after appointment. The following are the terms of engagement:—

1. Selected candidates to be termed "lady nurses."
2. Candidates to be between 28 and 35 years of age, and to produce certificates of birth.
3. To be examined by the President of the Medical Board (before engagement) as to fitness for service in India.
4. To produce certificates of at least three years' training at a hospital in which adult males receive medical and surgical treatment and in which a nursing staff is maintained. Also to furnish recommendation from the matron of the hospital at which they have been trained.
5. Engagement to be for not less than six months except in case of misconduct or break-down of health. Notice of one month to be given before termination of an engagement on the part of Government or the lady nurse.
6. Free passage by second saloon P. and O. Company's steamers to India and back; but resignation within a year except on account of ill-health not to entitle to a free return passage from India.
7. Pay, Rs. 175 per mensem; in addition to free quarters, fuel, lights, and punkah pullers; to commence from date of embarkation to India.
8. Outfit allowance of £10.
9. Travelling expenses from and to residence in England to port of embarkation (claims for conveyance of baggage to be supported by vouchers). Six hundredweight of baggage allowed.
10. To embark for India within one week after appointment.

### Nursing Appliances.

MESSRS. SOUTHALL'S EXHIBITION.

In this small exhibition, held at 74, Regent Street, are many interesting exhibits, among them the baskets and bags for the district nurse's use were especially noticeable. Both the bags and baskets have been fitted up by a nurse, and contain all that a nurse is likely to need. They are also made as light and as easy to carry as possible, and in general appearance, as well as in other respects, are sure to please their owners. Specimens of the knapkenettes used in the nursery of H.R.H. the Duchess of York are shown, and are of absorbent wool on one side and non-absorbent on the other. The accouchement sheets, chest protectors, &c., are made of the same material. A useful little electric bell and battery, which being small, light, and movable, would stand anywhere as most convenient, had a long cord attached, so that the patient need only press the knob, when the bell would ring to summons the nurse. The beef-tea extractor (Dolby's patent) supplies a great need. By the arrangement of the porcelain inner vessel, which is cut off from the outer vessel by an air chamber, the contents are prevented from heating over 120 deg. Fahr., thus allowing all the nourishing juices to escape from the meat. Messrs. Southall's cod-liver oil was on view, beside a carefully made model of the factory at Balstad, Lofoden, Norway, where it is made. The bed-pans, poultice-heater, rubber hot-water bottles, handy accident cases, hypodermic syringes (nickel-plated, which may be boiled), are all worthy of notice and convenient in use. The rubber hot-water bottles have the handle placed at one side of the neck and an escape tube for steam. The food warmers are compact little apparatuses with kettle, saucepan, and pannikin, spirit lamp, and a pan for night-light, which must be a great boon to all who need refreshment at awkward hours.

### Everybody's Opinion.

[Correspondence on all subjects is invited, but we cannot in any way be responsible for the opinions expressed by our correspondents. No communication can be entertained if the name and address of the correspondent is not given, or unless one side of the paper only is written on.]

UNSEEMLY AMUSEMENT.

We have received many replies to a letter from a correspondent of last week entitled "Unseemly Amusement," but we do not publish them because these communications so far are written under a misapprehension. The writer did not put forward objections to dancing as such for nurses, only she does not think that dancing in an institution devoted to the sick and dying seemly, especially in conjunction with the medical men who are associated with the nurses in the care of the suffering. We and all who have the health and happiness of nurses at heart are only too glad for them to have suitable opportunities to indulge in all innocent amusements.

BREAKING CONTRACTS.

J. Vincent Jackson writes: May I protest against a practice which I am afraid is becoming a little more general than is either creditable or desirable. In the columns of The Hospital there are generally a large number of advertisements offering situations either in hospitals, district nursing work, and nursing institutions, and this is an opportunity of great value to all concerned, and I am anxious that nothing should occur to lessen it or to impair its utility; but I am afraid that if a practice amongst nurses, which my experience leads me to say is growing, is not stopped, an injury will be done to this very convenient avenue for those who offer work to nurses, and for nurses who are desirous of obtaining good and approved nursing work. I am in a position to affirm that nurses seeking an appointment answer more than one advertisement; this may, perhaps, be inconvenient, but is not in itself objectionable. But some, after they have been selected for the position applied for, and have in writing accepted the appointment, and made arrangements for taking up the work, in the interval write and decline the engagement, on the ground that they have been offered better terms elsewhere. I hope, Mr. Editor, you will denounce this reprehensible practice, which is not only bad form, but is prejudicial to good faith and probity.

*** Our correspondent draws attention to an evil that unprincipled nurses bring upon the whole profession. A nurse acting in such a way as he describes is guilty of a breach of contract, and no doubt is liable before the law. If the sufferers would summon the culprits and demand damages they would undoubtedly be instrumental in improving the position of affairs. Whereas now all nurses share the suspicion of want of principle, then the guilty persons alone would have to bear the penalty.

### Where to Go.

The Guild of St. John the Evangelist.—Nurses of Huguenot descent are informed that the Huguenot Guild of St. John the Evangelist, Bloomsbury, incorporated with the Church Guilds Union for the Province of Canterbury, will attend the annual festival service in St. Paul's Cathedral on Wednesday evening, the 27th inst. It is possible that they may take an interest in being present on the occasion. Notice is also given that the next conversazione of the guild will be held on Tuesday evening, November 16th, at the Vestry Hall of St. George, Bloomsbury. Applications for tickets of admission should be addressed to the Incumbent, Church Vestry, St. John the Evangelist's, Bloomsbury, 233, Shaftesbury Avenue.

Royal British Nurses' Association.—The first sessional lecture of the season will be delivered on Wednesday, December 1st, at the offices, 17, Old Cavendish Street, W., by Sir John Lubbock, on the subject of "Ants."

### A Retrospect.

At the Cape of Good Hope hospitals are steadily working towards greater efficiency. Two difficulties, which are happily unknown in Europe, press for solution. They are the necessity of providing hospital accommodation for the coloured population and making provision for lepers. Much remains to be done on both scores before the requirements of hygiene and humanity are fulfilled.

With regard to nursing matters, the one-year trained nurse still exists, but a higher standard is being steadily sought after by the training schools attached to the different hospitals. The Albany General Hospital has converted the building formerly occupied by the medical officer into sleeping apartments for the nurses.

Several radical changes have taken place, principally at Kimberley Hospital. The St. Michael's Sisterhood, who for the last eighteen years had nursed the hospital in a perfectly satisfactory manner, severed their connection with it, and the board assumed the direct control of nursing affairs, and a lay nursing system has been substituted.

At the Queenstown Frontier Hospital arrangements have been made to enable probationers to train and take certificates.

At the Suburban Hospital, Woodstock, the training for probationers is only one year.

### Appointments.

### MATRONS.

Wallsend and Willington Infectious Diseases Hospital.—The newly-appointed Nurse-Matron of the above hospital is Miss Emma Wallace. This lady received her training at the General Infirmary, Northampton, and has since been engaged in private work, and also at the Peebles Joint Hospital.

Woolwich and Plumstead Cottage Hospital.—On September 22nd Miss Mary Harcourt Poulton was appointed Matron of this hospital. She received her training at the London Hospital, and has subsequently been night superintendent at St. Mark's Hospital, City Road, E.C. Miss Poulton holds the L.O.S. diploma.

Homerton Infirmary.—Miss Louisa Griffiths, for five years assistant matron at the Eastern Fever Hospital, Grove Road, E., was appointed Matron of this infirmary on October 13th. Miss Griffiths was trained at the Brownlow Hill Infirmary, Liverpool.

Hants Rural Nursing Association.—Miss Anne Phelps has been appointed County Superintendent of this association. She is a Queen's Nurse, and was trained at the London Hospital, and has held posts at Paddington and Camberwell.

Norwood Cottage Hospital.—On October 15th Miss Edith Fry was appointed Matron of the above hospital. She was trained at the London Hospital, and has been matron for five and a half years at the West Cornwall Miners' Hospital, Redruth, Cornwall.

Homerton Infirmary.—On October 13th Miss Clara E. Williams was appointed Assistant Matron of this infirmary. Miss Williams was trained at the General Infirmary, Birmingham, and was subsequently staff nurse and sister-in-charge of the same institution.

Fever Hospital, Mountain Ash.—Nurse Jones, from Cardiff Sanatorium, was appointed Matron of this institution on September 20th. She was trained at Manchester, and afterwards became charge nurse at the City Hospital, Liverpool.

Devon and Exeter Hospital.—Miss Florence Pepper, trained at St. Thomas's Hospital, London, and subsequently ward sister and temporary assistant matron of the same hospital, became Lady Superintendent of the Devon and Exeter Hospital on October 14th.

### Novelties for Nurses.

### SPECIALITÉS FOR NURSES AT MESSRS. DEBENHAM AND FREEBODY.

It would seem as if ingenuity had come to an end in the matter of fresh devices for nurses' uniforms, but such is not the case, as a visit to Messrs. Debenham and Freebody's well-known emporium testifies. Celebrated, and justly so, for the elegance and good taste of their designs, they have recently brought out an improved cloak in the Princess style, which is likely to become a great favourite among nurses. It is semi-fitting to the figure, and very graceful in appearance and style. The Dora is another shape not quite so new, but in no danger of losing its popularity. It fits into the waist at the back the sides and fronts hanging loose like a circular. The price of this cloak is only 33s. 6d., and is supplied in any colour, though black or navy are most usually asked for. We are glad to observe an excellent cycling model, which will be found a real boon to district nurses, who nowadays have to add the accomplishment of the wheel to their other qualifications. The Victoria, as it is called, is composed of skirt and upper part in one, buttoned down the front, and at the side. A short cape covers the arms and protects the shoulders. It can be had in any colour, price 55s. There are some charming bonnets now on view. The Adelaide and the Dorothy are both neat shapes trimmed with velvet or ribbon, and finished off with a white ruche under the brim. In caps the Mildred is very becoming. It is made of white jaconet and drawn into position by a string at the back, and has a deep upstanding frill round the front of a coronet shape. White strings tie in a neat little bow under the chin. In addition to what we have just described there is a wide and varied assortment of lingerie. Linen cuffs and collars, Irish cambric handkerchiefs in all qualities, and many delightful garments for underwear in the softest and purest woollen material. Travelling bags filled with every requisite, instruments of all sorts, chatelaines and wallets, all have their place in the nursing department at Wigmore Street, to which we are glad to note the welcome addition of furniture suitable for nurses' bedrooms. For durability and elegance of design we have no hesitation in stating that Messrs. Debenham and Freebody have few, if any, rivals in the market. The prices are marvellously reasonable, and any establishment about to furnish would be well repaid in making their selection from those we have just described above. Old bedsteads can be made as comfortable as those of the newest design by having them fitted with Messrs. Debenham and Freebody's admirable spring mattresses on wooden frames. In cases that we know of where the experiment has been tried it has succeeded beyond expectation. The proximity of a tea-room to this department will be, no doubt, gratefully recognised by the numerous visitors to whom shopping is apt to become monotonous as five o'clock comes near.

### Minor Appointments.

General Hospital, Walthamstow.—On October 11th Miss A. Childe, trained at Crumpsall Infirmary and holding certificates for medical, surgical, and midwifery nursing, was appointed Staff Nurse of the male adult wards of the above institution.

Noble's Isle of Man Hospital.—Miss Jane Fish, trained at Salford Royal Infirmary and afterwards charge nurse in the same, was appointed on September 1st Charge Nurse of the Noble's Isle of Man Hospital.

Burton-on-Trent.—Miss M. A. Griffiths, of Bristol Royal Infirmary, has been appointed Charge Nurse of the surgical ward at the Infirmary, Burton-on-Trent.

Infirmary Dispensary, Bolton.—Miss Jessie Knowles, who was trained at this infirmary, has been appointed Sister of female wards.

### For Reading to the Sick.

LIFE'S RESPONSIBILITY.

**Verses.**

AND much he knows, and much he thinks
And he is more than all he knows;
For still aspiring, still he drinks
Fresh inspiration as he goes;
More careful that the Man shall grow
Than that the mind should understand,
He loves all creatures here below,
And touches all with tender hand.

Only add
Deeds to thy knowledge answerable; add faith;
Add virtue, patience, temperance; add love;
. . . Then wilt thou not loath
To leave this Paradise, but shalt possess
A Paradise within Thee, happier far. —*Milton.*

Each hour has its lesson, and each life;
And if we miss our life we shall not find
Its lesson in another—rather, go
So much the less complete for evermore,
Still missing something that we cannot name,
Still with our senses so far unattuned
To what the Present brings to harmonise
With our soul's Past. —*H. H. King.*

Tell me not, in mournful numbers,
"Life is but an empty dream,"
For the soul is dead that slumbers,
And things are not what they seem.

Life is real! Life is earnest!
And the grave is not it's goal!
"Dust thou art, to dust returnest"
Was not spoken of the soul:

Not enjoyment and not sorrow
Is our destined end or way—
But to act that each to-morrow
Finds us farther than to day. —*Longfellow.*

**Reading.**

Everyone must do his best; he must pray his best; he must sing his best; he must attend his best. If we did all it would be little, not worthy of Him; we do little it will suffice to show our faith, and He in His mercy will accept whatever we can offer. He will accept what we prefer to give to Him to giving it to ourselves. When, instead of spending money on our own houses we spend it on His House; when we prefer He should have the gold and silver to our having it, we bring Christ nearer to us; we show that we are in earnest, evidence our faith.—*Dr. Newman.*

After doing anything we should frequently examine what has been the spirit of our actions; we should search whether we began them to please God and went on to glorify ourselves. We should see whether we can trace the little but easily distinguishable evidences which mark the inward swelling or restless itching of vanity; the liking, if not to talk with open and offensive vain glory, about ourselves and our doings, yet still to hover so near ourselves that we can make short flights back to self, our excellence, and our successes; or the saying what will lead others to speak well of us; or the listening with greedy ears to commendations when they come.—*Bishop Wilberforce.*

### Notes and Queries.

**The contents of the Editor's Letter-box have now reached such unwieldy proportions that it has become necessary to establish a hard and fast rule regarding Answers to Correspondents. In future, all questions requiring replies will continue to be answered in this column without any fee. If an answer is required by letter, a fee of half-a-crown must be enclosed with the note containing the enquiry. We are always pleased to help our numerous correspondents to the fullest extent, and we can trust them to sympathise in the overwhelming amount of writing which makes the new rules a necessity. Every communication must be accompanied by the writer's name and address, otherwise it will receive no attention.**

*Uniform for Cycling.*

(30) 1. Could you kindly give me any suggestions for a uniform which would be suitable for a district nurse to cycle in in all weathers? 2. Would a sailor hat be permittable?—*Puzzled.*

See "Novelties for Nurses" in this and last week's "Mirror." 2 There is no hard and fast rule, but a bonnet is more professional.

*Operation.*

(31) Can you tell me whether there is any hospital in London where anyone not a nurse can go to see the operations without even paying? Does the Royal Free in Gray's Inn Road allow this?—*Meta.*

No one except medical men and students and those engaged in the work going on can be present at an operation at any hospital without a breach of propriety.

*Army Nursing.*

(32) I wish to become an army nurse, and would be greatly obliged if you will tell me how and where I can receive the necessary training.—*Army Nurse.*

See Honnor Morten's book, "How to Become a Nurse," 2s. 6d., post free, from the Scientific Press, 28, Southampton Street, Strand.

*Monthly Training.*

(33) I am a monthly nurse, but I am anxious to become certificated. Will you please tell me if I must go through the same training as a beginner, how long it will take, and if I must pay?—*Isa Andrews.*

The training lasts from three to six months, according to the ability of the pupil and the school chosen. The East End Mothers' Home, 396, Commercial Road, E., gives excellent training at the minimum cost.

*An Asylum Attendant.*

(34) Will you kindly give me the address of the Berrywood Asylum mentioned in THE HOSPITAL, September 25th? I have been in an asylum ten months, but found the nurses there unlike those you mention at Berrywood, being (as I thought) mostly girls of giddy, unrefined natures, and I should like to finish my training as mental nurse.—*Ecila Lleb.*

Candidates from other asylums are not taken at Berrywood.

*Training.*

(35) Will you kindly reply to the following questions: (1) Are there any asylums or infirmaries in or near Southampton? I wish to get some training in a public institution, being at present an untrained but experienced mental nurse-attendant. (2) Under the heading, "Modern Sociology—Hereditary Neuroses in Children," in THE HOSPITAL of May 29th, a book by Dr. Marshall Hall is mentioned in a footnote. Who is the publisher, and is it a book suitable for one like myself, who wishes to study insanity, or could you recommend me any books? (3) Also in a later number of THE HOSPITAL, I have lost date, but under "Hereditary Neuroses in Children—V.," a work is mentioned in a footnote by Strahan, "Marriage and Insanity."—*Edith.*

(1) The Royal South Hants Infirmary, Southampton; matron, Miss Mollett. (2) Dr. M. Hall's "Observation in Medicine" is written for medical men. (3) Dr. Strahan's books are "Marriage and Disease" and "Suicide and Insanity," which can be obtained from the Scientific Press, at 6s. and 2s. 6d. respectively, post free.

*Fever Nursing.*

(36) 1. Can you tell me the duties of second assistant nurse in fever hospital. 2. If they give certificate to same? (3) Also is it necessary to be vaccinated since the age of 15 to enter same?—*M. F.*

Write for information to the matron of the fever hospital you propose entering. (2) Only for fever nursing. You should obtain your nursing certificate from a general hospital. (3) Certainly, if you intend to nurse small-pox; not otherwise. M. F. must remember that institutions vary in every way, so that it is impossible to lay down one rule for all.

*Epileptic Patients.*

(37) Is there any home where an epileptic young woman could be taken? It is a very poor case. (2) What is the address of the Colony for Epileptics?—*X Y Z.*

(1) You cannot do better than write to the Secretary of the National Society for the Employment of Epileptics, Chalfont St. Peter, Bucks. Offices, 12, Buckingham Street, Strand. See also index, Burdett's "Hospitals and Charities" for 1897, under Epilepsy.

*Home for Ayahs.*

(38) 1. Will you kindly give me the address of the London Home for Ayahs? I am nursing a lady who requires one to return to India with her in November. I have heard of the home, but am unable to find the address. 2. An answer by return will oblige.—*E. Jackson.*

We cannot find address of London Home for Ayahs, to which you refer. Although it is not connected with "nursing," we should be glad to know of it if you succeed in hearing of it. The shipping companies sometimes know of ayahs wishing to return, and advertisements occasionally appear in the *Morning Post.* We are sorry not to reply by return of post, but all queries requiring an answer by post must be accompanied by a fee of 2s. 6d.

